# Factors That Predispose Undergraduates to Mental Issues: A Cumulative Literature Review for Future Research Perspectives

**DOI:** 10.3389/fpubh.2022.831349

**Published:** 2022-02-16

**Authors:** Pierpaolo Limone, Giusi Antonia Toto

**Affiliations:** Learning Science Hub, University of Foggia, Foggia, Italy

**Keywords:** anxiety, depression, online learning, COVID-19, medical education, SAD, mental health, psychiatrist

## Abstract

Distress and mental health issues among college students is an emerging topic of study. The aim of this research work is to illustrate academic and social risk factors and how they prove to be predictors of anxiety and depressive disorders. The methodology used is a cumulative literature review structured over 10 systematic phases, and is replicable. Showing considerable potential for cumulative research, the relevance of this study reflects the concern of the academic community and international governments. The articles selected range from categorization of disorders in relation to mental health, to reporting the condition of rhinestones and difficulties of students in university contexts. In conclusion, the research focusses upon predisposing, concurrent or protective factors relating to the mental health of university students, so that institutions can act on concrete dynamics or propose targeted research on this topic.

## Introduction

Mental health and mental health-related issues have been a matter of concern for quite a long time earning little regard and interest from the respective healthcare facilities and systems. Governments have put inadequate measures to ensure that citizens' mental challenges are handled rightfully to achieve high levels of mentally healthy people. The perpetuated issue has also developed in various sectors of society. The current situation in a learning institution is worth raising eyebrows, and therefore it deserves serious attention. Most of the undergraduate students and yet to graduate college students depict high levels of mental illness among the students, thus, depicting discomfort of the students and the level of neglect the health sector is faced with. The majority of today's people who are suffering from mental issues in society constitute college and university students (1). College students endorse high rates of mental health problems. While many colleges offer on-campus services, many students who could benefit from mental health services do not receive car. Indeed, nearly half of students who screen positive for depression, for example, do not receive treatment (2).

Adverse consequences are synonymous with undergraduate students with mental distress. The victims are likely to experience challenges such as impaired functioning in cognition, substance abuse, poor performance in their school work, and learning disabilities. They are likely to abuse drugs such as tobacco, alcohol, cigarette smoking, and other hard drugs that impair normal body functioning (3). Most of these drugs are associated with various risk behaviors, depression, and anxiety (3). This suggests that emotional discomfort raises the likelihood of developing additional mental health issues. For this reason, the prevalence of mental illnesses among university students is higher compared to people in other environments. The situation is almost similar in most colleges since they are predisposed to similar conditions and forms of livelihood. This inherent condition puts the future generation, which is inherently composed of the schooling individuals, at more risk in line with mental health and other health conditions that may arise due to the mental disorders.

Several factors have contributed to the mental distress and discomfort associated with undergraduates. For instance, sex has a significant contribution to the mental illnesses that people experience in learning institutions. The prevalence of the conditions tends to be a notch higher among female students than male students (4). Some students lack interest in fieldwork which affects their mental health in the long run. Introvert students are also more likely to fall victims and students who face various social challenges such as poverty (4). Most of the learning institutions have tight schedules and continuous sequences of study, which affects the students' performance and their mental well-being. Challenges and the predisposing factors that affect the students are bound to result from their school environment or their history; therefore, the growth environment and interaction play a significant role in determining one's health. Some of the predisposing factors are avoidable, while others are accustomed and tied to the students. Therefore, it is prudent to come up with measures to ensure control and regulation of the inherent situation of the undergraduate students who make up the future and continuity of our current society.

### Common Mental Illnesses Among Undergraduates

Undergraduate students face many mental issues; however, the prevalence of some of the health conditions is a bit higher than others. Experts and researchers use terminology like “crisis” and “epidemic” to describe American college student's mental health issues today. Mood disruptions are only one of the many mental health problems that college students face. Suicide, addiction, and eating disorders are examples of significant issues (5). Although mental health specialists emphasize the need to talk about such concerns, students often regard these pressures as a typical livelihood in learning institutions. In other circumstances, individuals may be unable to seek help due to a lack of time, energy, will, or financial resources (5). It is, therefore, a challenge in coming up with a satisfactory solution to the challenges of problems. Drawing the students' goodwill and desire to have their mental issues fixed is also a challenge as some of them may feel shy or mentally healthy, and that there is no need to go through medication. Similarly, identifying the deserving students and coming up with radical measures to satisfactorily come up with a solution is also challenging since acquiring the required resources is quite expensive. However, solving the problem is arguably easy through addressing some of the major health conditions that most undergraduate students experience.

Below, we investigate some of the most common mental illnesses among college students, such as depression, anxiety, suicidal thoughts, eating disorders, and addiction.

Depression is a widespread chronic medical illness that can affect thoughts, mood, and physical health. It is characterized by low mood, lack of energy, sadness, insomnia, and an inability to enjoy life (6). Victims of the condition tend to develop varying episodes of discomfort and displeasure that destruct them from their normative activities. Students may grow poor performance and the inability to fit in with their schoolmates in co-curricular and curriculum activities. According to the ACHA's 2018 poll, 40% of American students had at least one significant depressive episode that same year (5). A person may also feel sad, hopeless, powerless, and get overwhelmed with life situations and challenges that one may be facing. Trouble in completing assignments, challenges in paying attention, and reading are also synonymous with depression among undergraduates (5). It might be challenging to spot these concerns in others since students often minimize or refuse to discuss issues that are bothering them.

In ICD-10, Generalized anxiety disorder includes anxiety neurosis, anxiety reaction, and anxiety state, but excludes neurasthenia. ICD-10 also proposes diagnostic criteria for research: (i) at least 6 months with prominent tension, worry, and feelings of apprehension about everyday events and problems; and (ii) at least four symptoms out of a list of 22 items, of which at least one item is from a list of four items of autonomic arousal (palpitations/accelerated heart rate, sweating, trembling/shaking, dry mouth).

Anxiety was identified as a significant student mental disorder by 61 percent of survey respondents in the University of Pennsylvania study published by Locke et al. (7). Anxiety disorder symptoms are frequently misdiagnosed as everyday stress or dismissed as someone overly concerned. Panic attacks might be misinterpreted as a medical ailment, like a tension headache or heart attack, depending on how your body responds to high amounts of specific chemicals (7). Since each person's symptoms present differently, what sighs the existence of anxiety to one person may not be similar in another (7). Consequently, the causes of anxiety differ from one person to another; however, some causes are common among campus students. For instance, stress, life experiences, genetics, and brain chemicals commonly cause anxiety in people (7). It, therefore, requires adequate measures of utmost keenness to ensure that the condition gets eliminated from the learners' livelihood.

The APA defines completed suicide as a self-injurious act that results in death and attempted suicide as a non-fatal, self-inflicted, potentially harmful act that is intended to result in death but may or may not result in injury (8).

Approximately 20% of university students in the United States were reported to be suicidal in 2018 (9). Therefore, it implies that the mental condition is rampant and makes up one of the major mental illnesses common among American students. According to the Los Angeles Times' Healstaff (10) report, teenagers and young adults record the highest suicide cases in America. Since the population inherently dominates the composition of the universities, it insinuates that undergraduate students register the highest number of suicide cases. Many students experience dissatisfaction and doubt, but these feelings can spiral out of control, leading some to consider suicide seriously. Suicidal ideation manifests itself in a variety of ways. Speech, temperament, and behavior are all examples of common warning indicators (10). Persons may describe themselves as stuck, burdening others, as if they have no reason to live and have no purpose to live. Suicidal ideation causes a wide range of emotions: anxiety, impatience, loss of interest in previously appreciated activities, shame, rage, and melancholy (11). People may engage in certain activities, such as giving up valued items, withdrawing from family and friends, unexpectedly visiting someone to say bye, and searching the internet for ways to commit suicide (11). They also may sleep poorly or excessively, act rashly, show anger, and increase their drug and alcohol use (11). Whenever one is seen with the symptoms, a bold and patient approach should help the victim seek medical attention from a psychiatrist and facilitate the healing process.

Eating disorders are a group of illnesses characterized by significant changes in one's eating habits and a preoccupation with a person's shape or body. Eating disorders (EDs), including anorexia nervosa, bulimia nervosa, and binge-eating disorder, constitute a class of common and deadly psychiatric disorders (12). The health conditions can entail binge eating and deprivation of food, which sometimes results in purging. According to 2018 estimates from the National Eating Disorders Association, 10–20% of female college students suffer from an eating disorder, with rates continuing to grow (13). Male students have a lower incidence rate of 4–10% (13). The typical eating disorders among undergraduates include bulimia nervosa, anorexia nervosa, and binge eating disorder. Emaciation is a specific symptom of anorexia nervosa, characterized by an excessive preoccupation with thinness, a disordered body image, and anxieties about gaining weight (14). Constant desires that occur at any time of day and lead to binge eating characterize binge eating disorder (14). This condition is frequently linked to low self-esteem and a negative body image. Bulimia nervosa is a form of binge eating condition characterized by recurrent and frequent bouts of eating abnormally large amounts of food, followed by compensatory behaviors such as purging, fasting, or excessive exercise (14). The symptoms and indications of eating preconditions differ from person to person and condition to condition, and many are dependent on the mental state of the person suffering from the problem (14). Many college students fail to seek treatment for their eating disorders since they do not have an awareness that they have one.

Alcohol and recreational substances are commonly used by college students, which can be troublesome (15). Addiction is a psychological or physical dependency pattern on one or more substances, characterized by strong cravings and substance abuse despite knowing risks and consequences (16). Alcohol is the leading cause of many disorders and deaths for campus students, while some abuse drugs to induce their studying habits (17). The recreational activities that undergraduates use alcohol and other drugs for result in addiction which causes many diseases. Besides alcohol, students also abuse marijuana, cocaine, ecstasy, and benzodiazepines (17). The dire need and desire to abuse drugs for various purported gains may lead to health complications resulting in death and body organs' failure.

### Mental Illness Prevalence Among Undergraduates

Mental health disorders are common among students, with a higher incidence than in the general population. Statistically, more than half of the students in American public universities suffer from depression and anxiety (18). Similarly, a poll of undergraduate students at Coventry University in the United Kingdom found that many students had suffered mental health disorders such as anxiety and depression in 2006 (19). Maser et al. (20) showed that the prevalence of mental health disorders such as anxiety and depression cases are a notch higher in medical school compared to the general non-student community of the same age, which supports these findings. Over the last two decades, these investigations have shown that the frequency of Seasonal Affective Disorder (SAD) amongst students has remained more significant than the general population.

SAD is not only common, but it is also persistent among students. Zivin et al. (18) found that more than half of students maintain their higher anxiety and sadness over time by performing a 2-year follow-up survey research and study of students. This phenomenon could be related to a lack of SAD therapy or the persistence of pre-existing risk factors.

## Methodology

The cumulative literature review, a new and rigorous research method, is divided into 10 phases (21):

1. Selection of key concepts (especially of independent and dependent variables and the relationships between them).
2. Creation of a search string (in addition to selecting the keywords, it is necessary to include and exclude the studies found through the criteria used). The final goal of this phase of the research is to find a manageable number of studies through the following procedure: (1) query two or three search engines or databases; (2) use keywords in combination with “and/or”; (3) use filters to manage the enormity of the results (comparison with a second researcher as in this study would be desirable).
3. Export of the results from the databases and a merging of all the transcribed results in the form of a bibliography on a single worksheet.
4. Selection of primary sources by eliminating duplicates and excluding irrelevant studies based on titles. This step is necessary to create a separate list of systematic reviews on the topic. It is also essential for drawing up a list of the individual choices of the cumulative review.
5. Verification of the secondary bibliography, by checking the bibliographies of all the included studies. Studies that cite primary sources, such as other systematic reviews on the topic, must therefore be included under the studies not found in the initial search.
6. Data extraction (produced by the reviewers' work), in which the characteristics of the selected studies are extrapolated. These characteristics include key variables, type of research project, context, results, year of publication, etc. The exclusion criteria are also cumulative; i.e., they are formulated on the basis of the time available and the studies retrieved from the databases.
7. Updating of the results on the basis of recent publications, which may prompt an update regarding the initial work carried out. This must be done before the conclusion of the cumulative review.
8. Verification by the second reviewer, who checks the included studies.
9. Writing of the last phase of the report.
10. Exercising due care in the publicization of the revision. This involves making the data collection work explicit within the format of the paper: keywords, extracted data, results, etc.

Each phase is illustrated below, retracing the steps taken to carry out the cumulative review, in order to make the study replicable.

In this study, research, which was based on the cumulative literature review model, followed the ten-phase model set out in the previous paragraph. Specifically, the following keywords were selected: mental disease, risk factors, university, students (phase 1). Scopus, WoS and Google Scholar were selected as search engines. The search yielded 797 results that were selected, based on comparison by researchers, using the following inclusion-exclusion criteria: inclusion of all literature reviews in the 2011–2020 period, related studies on risk factors toward mental disorders of university students (phase 2), with exclusion based on primary source titles. The raw research data was transcribed on a spreadsheet, in order to enable a global view of the studies located and to start the selection work (phase 3). The file was “cleaned up,” in order to remove duplicate contributions and create a second list of systematic reviews of the literature (*n* = 4), one book, and one book chapter (phase 4). In the first file named “primary sources,” significant studies were selected and placed based on the title. The cleaned file contained *n* = 33 papers. For the second file containing the systematic reviews of the literature, the secondary bibliography included was consulted, and the studies already present in the first file of the present research were eliminated. In this case, 67% of the studies had already been identified in the comparison of the cumulative literature reviews. It would be desirable to build a reliability index of the cumulative review that took into account this value, i.e., the degree of replicability of the systematic studies already conducted (phase 5). Construction of a grid (Table 1) was carried out, using the results of the research and data extraction by the researchers who analyzed the key variables (key variables, type of research project, context, results, year of publication, etc.), by selecting as reported in the table, only the fields relevant to the research (phase 6). The research carried out in the first months of 2021 has been updated with more recent publications that have introduced a surveys studies of mental illness among university students (effects of the COVID emergency in terms of physical, cognitive and relational consequences). On the basis of the inclusion-exclusion criteria, other (*n* = 2) papers were included (phase 7). The complete file, containing the studies considered significant for the purposes of the construction of this work, was analyzed by the second researcher, in order to avoid errors in the research (phase 8). Steps 9 and 10 resulted in the production of this research paper.

**Table 1:** Summary of the results.

**Paper**	**Type**	**Number of participants or studies**	**Themes**	**Year**	**Age of population**	**Main results**
Anakwenze and Zuberi (22)	Paper		Urban poverty, mental illness, crime	2013		Social disadvantages such as poor housing and poverty pose more risk of mental disorders among students
Chernomas and Shapiro (23)	Paper	437	Stress, depression, anxiety, nursing students, clinical practice performance	2013	Adult	Nursing, medical and health-related students have a greater prevalence of depression and anxiety
Fares et al. (24)	Review		Stress, burnout, preclinical medical students, solutions to stress and burnout	2016	Adult	Medical and nursing students experience higher anxiety and despair
Flatt et al. (13)	Paper	3,516	Comparing eating disorder, athletes and non-athletes	2021	13–24	10–20% of female college students suffer from an eating disorder, with rates continuing to grow. Male students have a lower incidence rate of 4–10%
Grant and Chamberlain (25)	Paper	576	Family history of substance use disorders, vulnerability toward addiction	2020	18–29	Growing up in a challenging environment or being abused by a parent or relative raises the risk of getting depression or anxiety
Ghodasara et al. (26)	Paper	301	Medical students, mental health disorders	2011	Adult	Insufficient sleep causes stress due to poor academic performance, as sleep quality and quantity are linked to academic performance
Hassanzadeh et al. (27)	Paper	4,763	Iranian adults, stressors, psychological problems	2017	36.58 ± 8.09	Students suffering from academic stress are likely to perform poorly in their schoolwork
Healstaff (10)	Paper		Suicide, teens and young adults, highest on record	2019	Teen and adult	Teenagers and young adults record the highest suicide cases in America
Hersi et al. (3)	Paper		Mental distress, university students	2017		The intake of drugs, tobacco, alcohol, and smoking impairs normal functioning of the body and is associated with various risk behaviors, depression, and anxiety
Ishii et al. (28)	Paper	203	University students with mental disorders, dropping out, social maladjustment	2018	Adult	Receiving worse grades during their studies can have a severe impact on student's mental health, leading to the development of SAD
Joseph (5)	Paper	610,000	College students, suicidal thinking, depression, self-injury	2019	Adult	Trouble in completing assignments, challenges in paying attention, and reading are also synonymous with depression among undergraduates
Jochman et al. (29)	Paper	149	Mental health outcomes of discrimination, college students, racially diverse	2019	Adult	Discriminated individuals end up with low self-esteem and confidence
Karch (17)	Book		Pharmacological, medical, and legal aspects of drugs	2019		Alcohol is the leading cause of many disorders and deaths for campus students, while some abuse drugs to induce their studying habits
Kenney and Müller (30)	Paper		Environmental epigenetics, maternal care, biosocial life	2017		Stress caused by mental health issues in great-grandparents, grandparents, or parents changes one's DNA, making them more vulnerable to difficulty
Kim (31)	Paper	393	College students, ego, mental health	2013	Adult	Trauma, brain injury, chronic illness, drug and alcohol use can inhibit health to the college level and cause mental disorders
Lee et al. (32)	Paper	384	Mental health, coping, medical students	2012	adult	Mastery of the subject has been shown to negatively affect anxiety, self-esteem, and depression among college and university students, with those who have a mastery of the subject displaying less stress and anxiety
Loades et al. (33)	Review	51,576	Social isolation, mental health, children and adolescents, covid-19	2020	15.3 average age	Young adults, who make up the undergraduate population, are prone to experiencing high depression rates, which can also cause anxiety
Locke et al. (7)	Chapter		Psychological issues, students, counseling centers	2016		Anxiety was identified as a significant student mental disorder by 61 percent of survey respondents
Kawase et al. (34)	Paper	273	Undergraduate students, mindfulness, health condition	2008	Adult	Students majoring in psychology and philosophy, like medical and nursing students, are more likely than others to acquire depression during their studies
Maser et al. (20)	Paper	4,613	Medical students, psychological distress, mental illness	2019	Adult	The prevalence of mental health disorders such as anxiety and depression cases are a notch higher in medical school compared to the general non-student community of the same age
Macaskill (35)	Paper	1,197	Psychiatric symptoms, university students	2013	Adult	Not all research discovered a link between the study's subject and the development of SAD
Mofatteh (36)	Review		Risk factors, stress, anxiety, and depression, university undergraduate students	2021	Adult	Students who do not live a healthy lifestyle may experience shame, which can exacerbate their SAD symptoms
Rosenthal et al. (37)	Paper	412	Female college students, alcohol consequences, predict major depression onset	2018	Adult	In students, there may be certain negative behaviors associated with alcohol consumption
Scholz et al. (38)	Paper	163	Dentistry students, mental risk factors, enhancement of mental health	2016	Adult	Both the cases of students suffering from mental problem symptoms and the severity of their SAD increase during test time, indicating a direct link between academic stress and students' psychological health states
Schweizer et al. (39)	Paper	2,544	Subjective memory complaints, symptoms of depression, memory performance in affective contexts	2018		There are chances that depression and other related disorders such as momentary memory loss and lack of concentration are causes of bad academic marks
Stallman (40)	Paper	6,479	University students, psychological distress, general population data	2010	Adult	Grades and mental health can have a reciprocal relationship, with poor mental health causing students to receive poorer grades
Skilbred-Fjeld et al. (41)	Paper	4,571	Cyberbullying, mental health, late adolescents	2020	18–21	Contemporary cyberbullying is also characterized by imposing anxiety, low self-esteem, and depression among young adults
Stasak et al. (11)	Paper	244	Read speech voice, individuals with recent suicidal ideation or suicide attempt	2021		Suicidal ideation causes a wide range of emotions: anxiety, impatience, loss of interest in previously appreciated activities, shame, rage, and melancholy
Soh et al. (4)	Paper		Medical students, mental distress, housing and travel time	2013	Adult	The prevalence of the conditions tends to be a notch higher among female students than male students
Tavolacci et al. (42)	Paper	1,876	University students, perceived stress, substance use, behavioral addictions	2013	Adult	Students who felt supported by their university were less stressed and were less likely to engage in substance abuse, demonstrating the importance of social support in preventing and treating depression symptoms
Turner et al. (19)	Paper		University students, mental health problems, Ethnic minority students	2007	Adult	Many students had suffered mental health disorders such as anxiety and depression in 2006
Wade et al. (14)	Review		Eating disorders, on the incidence, prevalence and mortality rates	2011		The symptoms and indications of eating preconditions differ from person to person and condition to condition, and many are dependent on the mental state of the person suffering from the problem
Wallace et al. (43)	Paper	440	College students, sleep, health	2017	Adult	Students in the United States frequently report significant stress levels and inadequate sleep
Younghans (9)	Paper	67,000	College students, thoughts of suicide	2018	Adult	Approximately 20% of university students in the United States were reported to be suicidal in 2018
Zou et al. (16)	Review		Substance and non-substance addiction, definitions, biological and psychological underpinnings	2017		Alcohol and recreational substances are commonly used by college students

## Results

It is clear from the selected articles that the idea present in the introduction is confirmed: the highest number of studies were on mental disorders, as well as on alimentary disorders. On the contrary, technology and other new addictions among university students are still little studied. They were therefore excluded from this study (Table 1).

### Risk Factors

From the analysis of the literature, several risk factors emerge that are involved in the development of mental problems in students. In particular, it is possible to classify the main factors in: academic factors, social factors, psychological risk factors, lifestyle factors and physiological factors.

Among the academic factors, the inverse correlation between time spent in study and poor results emerges. Academic results are, in fact, correlated with job placement and other higher education programs. It follows that, at times, this can be detrimental to students' mental health. In addition, elements such as loneliness and social isolation can often induce worry and melancholic states that play a major role in learning. One example is the pandemic situation that has forced millions of students into a scarcity of relationships for a very long time. Additional pivotal factors are those of a psychological nature: disappointments, stress, and perceived anxiety can have a major impact on academic performance; in addition, abuse and mistreatment negatively affect cognitive, emotional, and social development.

The period of change characterized by entry into college often involves changes in lifestyle as well: there may be a tendency to increase intake of drugs, alcohol, and various substances that, if abused, can alter functioning patterns. One of the pivotal factors, however, is the biological makeup of the individual: genetic history and health status have high implications in mental health.

#### Academics Factors

SAD can get caused by a variety of university-related academic pressures. The degree's subject is one of these strongly prevalent factors. When compared to their non-medical colleagues, nursing, medical and health-related students have a greater prevalence of depression and anxiety (23). Medical and nursing students, who have both theoretical and patient-related responsibilities, typically have an enormous workload among undergrads and, as a result, experience higher anxiety and despair (24). Furthermore, students majoring in psychology and philosophy, like medical and nursing students, are more likely than others to acquire depression during their studies (34). Medical and nursing students who work with people's health may develop melancholy and anxiety due to their worries about making mistakes that could hurt them or their patients (23). Students whose degrees include practical components may travel to new locations for fieldwork and job experience, adding to their anxiety and stress (23). However, it's essential to determine whether students with underlying mental health issues are more prone to pick disciplines like philosophy or psychology or subjects that lead to caring careers like nursing and medicine.

Furthermore, some prospective students, particularly those studying nursing and medical, often lack explicit knowledge of the workload and curriculum associated with their field of study before enrolling in university, and as a result, they may become disillusioned once they begin their studies (23). It's worth noting that not all research discovered a link between the study's subject and the development of SAD (35). Variances can explain this phenomenon in sample type and size, resulting in disparities in workload and curriculum, such as courses taught in different universities across the world.

Studying for a degree at the university level can be a challenging endeavor that necessitates mental work. Mastery of the subject has been shown to negatively affect anxiety, self-esteem, and depression among college and university students, with those who have a mastery of the subject displaying less stress and anxiety (32). Additionally, students studying in a foreign environment where there is a use of a non-native language sigh high levels of anxiety and sadness during their freshman year, with their stress levels decreasing with time (32). This is due to the fact that students studying in a foreign language are typically people who have moved abroad and thus take a while in adjusting to their new form of livelihood. Domestic and international students' depression and anxiety levels can be linked to the year of study, with newcomers entering university. On the other hand, final-year students experience the highest levels of anxiety and depression, and various risk factors (32). First-year students experience SAD due to difficulties adjusting to university life, negative family experiences in the past, social isolation, and a lack of friends. Final-year students report unpredictability about their years ahead, prospective work opportunities, university debt repayment, and adjusting to life after school as major risk factors for SAD (32). As a result, as students go through their degrees and learning process, there is a change in SAD potential risk themes.

Students spend a large percentage of time engaged in academic pursuits at university, and poor academic performance can harm their mental health. Receiving worse grades during their studies can have a severe impact on student's mental health, leading to the development of SAD (28). Academic achievement throughout undergraduate education can influence degree categorization, affecting students' opportunities, including job placement or entrance to postgraduate programs. On the other hand, both the cases of students suffering from mental problem symptoms and the severity of their SAD increase during test time, indicating a direct link between academic stress and students' psychological health states (38). However, there is no direct correlation that is well-established. There are chances that depression and other related disorders such as momentary memory loss and lack of concentration are causes of bad academic marks, or that students get anxious and depressed due to their poor exam performance (39). Grades and mental health can have a reciprocal relationship, with poor mental health causing students to receive poorer grades (40), creating a vicious loop of academic performance and mental health. Interestingly, students' social connection and coherence to the campus community during exam periods decreased (38). This phenomenon can be explained by students' lower participation in university social events and clubs and a higher sense of competitiveness among their peers. Furthermore, students interact with lecturers, instructors, tutors, and other staff members both directly and indirectly; as a result, the interaction between academic staff and students can impact students' mental health. Another factor that contributes to SAD among undergrads is a bad and abusive interaction with teachers and mentors.

Part-time students are more likely to be emotionally stable and free from mental illnesses compared to full-time students. Students enrolled for part-time studies are more likely to be employed, and therefore they have a constant flow of income. Similarly, they are less likely to experience some social predisposes that may induce mental illnesses due to their schedule. Unlike full-time students, they are free-wheel and do not have a limited and timed duration to complete their courses. Their financial advantage puts them in a better position; however, they are also likely to experience other forms of predisposing factors. The negative predisposes that are more likely to cut across all students, for this reason, include the pressure accrued from school workload, phobia of performing poorly. They also entail the wrong expectations built on the courses and institutions of learning, a student's year of study, poor relationship with the staff with which a student interacts at the university.

#### Social Factors

In human livelihood, everyone is exposed to society and that an individual and society are two inseparable entities. Society has a significant influence on a person's thought ideology and self-actualization. Naturally, a person's description and identification of oneself gets determined by society. For this reason, society dramatically influences a person's state of mental health. Whenever a person coexists with others in a relatively fair environment or at par with the majority fortunate, the individual's mental health state is likely to be boosted. The case is dissimilar when a person belongs to a few unfortunate members of the community. Some of the social predisposes are therefore likely to perpetuate disorders in undergraduate students or otherwise breed them.

Loneliness and social isolation are a matter of concern among students, especially due to the advent of online learning, which discourages interaction. In modern society, especially after the COVID-19 pandemic, most institutions of higher learning have adopted online learning to facilitate the continuity of education and the learning process. This form of learning has hindered students' possibility of interaction with their peers in a classroom environment. This phenomenon has perpetuated the inherent social condition and situations of some students, especially introverts. According to Loades et al. (33), young adults, who make up the undergraduate population, are prone to experiencing high depression rates, which can also cause anxiety. The isolation and limitation of interaction among the young population require mitigation to ensure that the issue gets resolved at the early stages of inception (33). Self-solation and loneliness are also associated with having few friends, thus putting one at risk of experiencing mental illnesses in college. More often than not, loneliness can lead to low self-esteem and confidence, which breeds anxiety.

Social disadvantages such as poor housing and poverty pose more risk of mental disorders among students. Among learners, poverty is associated with poor performance in school in line with behavior, cognition, and attention-related issues (22). Therefore, it is associated with anxiety, schizophrenia, depression, delinquency, and other mental health disorders that are synonymous with young adults. Additionally, poverty increases one's risk of getting traumas and abuse, especially during childhood, and losing crucial family members (22). High-income inflow in a home setup reduces chances and risk of domestic violence. It, therefore, goes without saying that when the condition is otherwise, the students are likely to get exposed to unbearable environments at home, which yields mental conditions. Similarly, students coming from poor backgrounds have instilled internal pressure and desire to evict themselves from poverty. The fear of poverty and the desire to become wealthy gives students discomfort and pressure since they always think that it is likely to cost them severely (22). Students who live in poor housing facilities are also likely to develop low self-esteem and confidence. They view themselves as inferior to other classes of students (22). Other students may also discriminate and underrate them, thus brewing mental conditions that are stringent and adverse. Therefore, it is wise and socially acceptable that students should not let their social situation of poverty and poor housing ruin their idea and sense of self-esteem and confidence.

Bullying and social discrimination impose mental health conditions that may affect the students' performance and cause long-term health conditions. Bullying, especially in the school environment, affects both the victims and the perpetrators in different ways (41). It may cause trauma, behavior, and bodily implications and affect one's identity. Contemporary cyberbullying is also characterized by imposing anxiety, low self-esteem, and depression among young adults (41). The psychological discomforts and distress may yield a personal thought toward a person, thus harming oneself. The individuals are likely to behave in a manner that can trigger suicide attempts and other forms of self-harm (41). As a result of low self-esteem, a person may also become an introvert, thus interfering with one's potential to interact with other people. Perpetrators are likely to have interaction problems and the inability to socialize with their fellow students since they have instilled fear. The situation is almost similar when one experiences various forms of discrimination. Discriminated individuals end up with low self-esteem and confidence, as well as the desire to rise above their perpetrators (29). This state breeds anxiety and depression among the victims.

#### Psychological Risk Factors

University and college students also get exposed to various psychological stressors and displeasures that negatively impact their mental health and performance. Some social predisposes are also likely to cause psychological discomfort and resulting mental illnesses in a university or college setup. Some early childhood preconditions are also likely to impact a person psychologically, even at the tertiary level of education (44). For instance, childhood trauma, abuse, and neglect are likely to be more disastrous when a person reaches the university or college level. Trauma greatly impacts a person's thoughts and feelings about oneself and how they relate with other people in society. Students, especially females, who have gone through a traumatic experience are likely to develop mental illnesses and conditions such as post-traumatic stress disorder (PTSD), depression, or anxiety (45, 46). Childhood maltreatment has a negative impact on cognitive, social development, and emotional development leading to problems with interaction and communication, as well as making people more prone to negative emotions in general and noticeable behavior problems like emotional maladjustment and anxiousness, hyperactivity, antisocial traits, and delinquent behaviors (45). Mistreatment during childhood is also likely to cause poor emotional intelligence, inhibited until college or university. Social support and refraining from mistreatment lead to mitigation of long-term adverse conditions such as depression and emotional self-regulation among children. Whenever the mitigation measures are not implemented, the victims are affected in adulthood. The instances are more severe among university and college students.

Long-term and severe stress is synonymously associated with causing mental illnesses among graduates. When stress becomes overwhelming and prolonged, the risks for mental health problems and medical problems increase. Long-term stress increases the risk of mental health problems such as anxiety and depression, substance use problems, sleep problems, pain, and bodily complaints such as muscle tension. Research indicates that stressful events cause significant psychological such as anxiety, distress, and depression (27). Similarly, severe and long-term academic stress leads to loss of welfare of the victims. Students suffering from academic stress are likely to perform poorly in their schoolwork (27). Poor performance perpetuates stress in the long run, as many students are accustomed to fearing academic failure and poor performance. Undergraduates may also get challenged by stressful life instances, such as breaking the law, which can cause mental discomfort and disorder. Its severity is also likely to cause other health conditions such as hypertension and asthma. It is, therefore, a predisposing factor that may inherently dominate a person's livelihood in the university.

Poor performance in school work leaves undergraduate students in thought which breeds mental illnesses. Whenever one performs poorly, there are chances that the person will get challenged mentally and develop the desire to work toward changing their results. However, failure for the same can cause a mental disorder due to the inherent academic expectations a person may develop. Similarly, mental illnesses affect a student's performance; therefore, the two risk factors are reversible, hence pausing the risk of cycle perpetuation. Attention to the students performing poorly in colleges and universities is essential in ensuring the cases of mental ill-health and continual unfolding of situations causing a cycle is fixed. This motive will help improve the learners' performance and work toward preventing some mental conditions that are likely to be incurred due to poor academic performance.

#### Lifestyle Factors

Moving away from family and starting a new life necessitates adaptability and flexibility for one to acclimatize to a new way of life. Most undergraduate students change their behavior and lifestyles as they leave their family setting and start a new life alongside their colleagues, friends, and classmates. SAD can be influenced by various lifestyle factors like alcohol intake, tobacco use, food habits, fitness, and drug usage. Students with mental problems consume a lot of alcohol (26). Alcohol is the most abused by undergraduates. It is synonymous with a series of mental disorders that they face. Alcohol is also addictive, and that when students overuse it, they are likely to experience various addiction disorders.

Another risk factor linked to SAD is tobacco smoking. It is widespread among students, particularly those from Eastern developing and developed nations like Japan, China, and South Korea (47). As a result of social bonding, many of the learners, especially male undergraduates, smoke, and the rate of social smoking is directly connected with SAD (47). Social smokers are less likely to give up their habit and are more likely to continue doing so, resulting in long-term detrimental psychological and physical health implications (47). Another key component in mental health among young individuals is illegal substance misuse (36). Academic stress and the social milieu in university dorms and student housing can lead students to take illegal drugs, smoke cigarettes, or consume excessive amounts of alcohol as a coping strategy, causing mental disorders (42). Students who felt supported by their university were less stressed and were less likely to engage in substance abuse, demonstrating the importance of social support in preventing and treating depression symptoms (42). It is especially important since a new social behavior or habit formed early in life might persist for a long time. Additionally, students who do not live a healthy lifestyle may experience shame, which can exacerbate their SAD symptoms (36). Rosenthal et al. (37) discovered negative behaviors associated with alcohol consumption, such as missing the next day's class, careless actions, self-harm, physical fight or verbal argument, the indulgence of unwanted sexual acts, shame, and regrets. The quantity of alcohol consumed can be the cause of depression and anxiety.

In universities and colleges, graduates adopt diverse sleeping habits that may yield mental illnesses and disorders. Many young people do not get enough sleep, causing sleep deprivation, a serious risk factor for depression and low mood (37). Students in the United States frequently report significant stress levels and inadequate sleep (43). The majority of undergraduates strive for academic brilliance, financial security, and the preservation of their lifestyle, which leads to poor sleep. Inadequate sleep can create a vicious cycle in which academic stress causes sleep deprivation. Insufficient sleep causes stress due to poor academic performance, as sleep quality and quantity are linked to academic performance (26). In general, poor sleeping habits are linked to lower learning ability, anxiety, and stress, leading to depression. Inadequate sleep, therefore, is likely to perpetuate a person's mental illness or otherwise fuel its inception.

In contrast with the predisposing factors, engaging physical exercise among students in colleges and universities is essential in protecting against mental dysfunctions. Students who claim to have limited time and fixed schedules may fail to engage in physical exercise and workouts. The development of SAD symptoms characterizes such students. Engagement in physical exercise and workouts makes the mind occupied and can also free off one's thoughts, which may cause mental illnesses. It also increases a person's interaction and enhances the social capabilities of interaction, which helps prevent some conditions. Physical exercise is also a form of therapy that requires one to exert physical exercise on the activity.

#### Physiobiological Factors

Physiobiological factors entail the factors that get affected directly and are related to the victim's biological composition, genetic history, and other health factors. For example, the mental health of an individual is inseparable from the family's history. Common disorders tied to an individual's family history include bipolar disorders, schizophrenia, dementia, depression, and anxiety (25). The genetic makeup determines the vulnerability of a person toward mental issues (25). People whose predecessors are associated with a certain mental illness are more likely to experience the same based on their genetic composition. Similarly, if one's family has a history of mental illness, one has likely been exposed to stressful conditions at some point in life. Growing up in a challenging environment or being abused by a parent or relative raises the risk of getting depression or anxiety (25). Epigenetics habits can also alter a person's emotions and habits, influencing people's biological composition, and it is likely to get passed to the next generation (30). Stress caused by mental health issues in great-grandparents, grandparents, or parents changes one's DNA, making them more vulnerable to difficulty (30). Furthermore, if a person's ancestors ate bad diets, had exposure to environmental pollutants, living with chronic stress, or did not receive proper prenatal nutrition, their genes—and thus an individual's—got altered, making them more likely to show mental illness health disorders.

Other biological factors such as pregnancy and birth complications, brain injury, chronic diseases, alcohol consumption, and drug abuse, as well as poor nutrition, are likely to predispose a victim to mental conditions. Some students have a history of complications during birth. Such students may inhibit the health conditions till the university or college level of study, and they are likely to cause mental disorders (31). Brain trauma and injury are also significant factors that may cause disorders in undergraduates. Some students have chronic illnesses such as diabetes and cancer, which expose them to discrimination, depression, anxiety, and low self-esteem. The diseases may also cause brain impairments, leaving the students mentally unwell (31). Usage of drugs and alcoholic drinks also influences the health status of an individual. Some drugs, such as marijuana, are associated with paranoia, resulting in adverse mental illnesses (31). Too much usage of drugs can also impair a person's eating habits which affect the learner's nutrition. It is, therefore, yields various eating disorders.

## Conclusion

Mental health-related issues and social well-being predisposing factors are a matter of concern in the community, especially among undergraduates. The prevalence of mental disorders is a notch higher among college and university students, raising the alarm on establishing some of the causes of the phenomenon. The predisposing factors include social, psychological, biological, lifestyle-based factors and academic factors. Academic excellence pressure and exerts various emotional feelings among students. The emotions and failure to meet their expectations land students into mental conditions that may perpetuate for a while. Change of environment and desire to adjust to a new form of livelihood in the university also causes a resultant change in lifestyle. More often than not, students commence drug and substances abuse which puts them at risk. A person's history of the family's genetic composition, chronic illnesses, and injuries of the brain also causes brain challenges (48). Interaction and other socio-economic factors are also crucial to a student's mental health that, when neglected, may result in disorders. Therefore, it is wise for the community to make haste and limit instances of the unfolding of the predisposing factors to achieve high standards of mental health among the undergraduates. This move will help in creating a future society that is mentally healthy.

## Author Contributions

PL: introduction. GT: methodology and conclusion. Both authors contributed to the article and approved the submitted version.

## Conflict of Interest

The authors declare that the research was conducted in the absence of any commercial or financial relationships that could be construed as a potential conflict of interest.

## Publisher's Note

All claims expressed in this article are solely those of the authors and do not necessarily represent those of their affiliated organizations, or those of the publisher, the editors and the reviewers. Any product that may be evaluated in this article, or claim that may be made by its manufacturer, is not guaranteed or endorsed by the publisher.
